# Genome based analysis of type-I polyketide synthase and nonribosomal peptide synthetase gene clusters in seven strains of five representative *Nocardia* species

**DOI:** 10.1186/1471-2164-15-323

**Published:** 2014-04-30

**Authors:** Hisayuki Komaki, Natsuko Ichikawa, Akira Hosoyama, Azusa Takahashi-Nakaguchi, Tetsuhiro Matsuzawa, Ken-ichiro Suzuki, Nobuyuki Fujita, Tohru Gonoi

**Affiliations:** Biological Resource Center, National Institute of Technology and Evaluation (NBRC), Kisarazu, Chiba, 292-0818 Japan; NBRC, Shibuya-ku, Tokyo, 151-0066 Japan; Medical Mycology Research Center (MMRC), Chiba University, Chuo-ku, Chiba, 260-8673 Japan

**Keywords:** *Nocardia asteroides*, *Nocardia otitidiscaviarum*, *Nocardia brasiliensis*, *Nocardia farcinica*, *Nocardia cyriacigeorgica*, Genome sequence, Type-I polyketide synthase, Nonribosomal peptide synthetase

## Abstract

**Background:**

Actinobacteria of the genus *Nocardia* usually live in soil or water and play saprophytic roles, but they also opportunistically infect the respiratory system, skin, and other organs of humans and animals. Primarily because of the clinical importance of the strains, some *Nocardia* genomes have been sequenced, and genome sequences have accumulated. Genome sizes of *Nocardia* strains are similar to those of *Streptomyces* strains, the producers of most antibiotics. In the present work, we compared secondary metabolite biosynthesis gene clusters of type-I polyketide synthase (PKS-I) and nonribosomal peptide synthetase (NRPS) among genomes of representative *Nocardia* species/strains based on domain organization and amino acid sequence homology.

**Results:**

Draft genome sequences of *Nocardia asteroides* NBRC 15531^T^, *Nocardia otitidiscaviarum* IFM 11049, *Nocardia brasiliensis* NBRC 14402^T^, and *N. brasiliensis* IFM 10847 were read and compared with published complete genome sequences of *Nocardia farcinica* IFM 10152, *Nocardia cyriacigeorgica* GUH-2, and *N. brasiliensis* HUJEG-1. Genome sizes are as follows: *N. farcinica*, 6.0 Mb; *N. cyriacigeorgica*, 6.2 Mb; *N. asteroides*, 7.0 Mb; *N. otitidiscaviarum*, 7.8 Mb; and *N. brasiliensis*, 8.9 - 9.4 Mb. Predicted numbers of PKS-I, NRPS, and PKS-I/NRPS hybrid clusters ranged between 4–11, 7–13, and 1–6, respectively, depending on strains, and tended to increase with increasing genome size. Domain and module structures of representative or unique clusters are discussed in the text.

**Conclusion:**

We conclude the following: 1) genomes of *Nocardia* strains carry as many PKS-I and NRPS gene clusters as those of *Streptomyces* strains, 2) the number of PKS-I and NRPS gene clusters in *Nocardia* strains varies substantially depending on species, and *N. brasiliensis* strains carry the largest numbers of clusters among the species studied, 3) the seven *Nocardia* strains studied in the present work have seven common PKS-I and/or NRPS clusters, some of whose products are yet to be studied, and 4) different *N. brasiliensis* strains have some different gene clusters of PKS-I/NRPS, although the rest of the clusters are common within the *N. brasiliensis* strains. Genome sequencing suggested that *Nocardia* strains are highly promising resources in the search of novel secondary metabolites.

**Electronic supplementary material:**

The online version of this article (doi: 10.1186/1471-2164-15-323) contains supplementary material, which is available to authorized users.

## Background

Actinomycetous strains of the genus *Nocardia* usually live in soil or water and play saprophytic roles in the environment, but also are opportunistic human pathogens, infecting the respiratory tract, skin, brain, and other organs of both immunocompromised and immunocompetent patients. To date, more than 80 species have been established in the genus *Nocardia*, and approximately one-third to one-half of the species have been reported as human pathogens [1–3]. Because of their medical importance, *Nocardia* strains have accumulated in microbial collections as a resource for clinical and scientific studies in the last few decades (e.g., [4–7]).

Although *Nocardia* strains belong to the Order *Actinomycetales* together with *Streptomyces* strains, the latter being known as a rich resource for discovery of secondary metabolites, few studies have been focused on secondary metabolites and their synthetic genes in *Nocardia* strains.

Type I polyketide synthase (PKS-I) and nonribosomal peptide synthetase (NRPS) gene clusters are two of the major secondary metabolite-producing clusters in bacteria and are involved in the biosynthesis of polyketide chains and nonribosomal peptides, respectively. It has been found that these clusters produce several medically and industrially important compounds, such as pathogenic factors, avermectin, erythromycin, and vancomycin.

In the present paper, we searched for PKS-I and NRPS genes in the genomes of representative *Nocardia* strains and analyzed their sequence similarities and differences in domain/module structures. While we were sequencing and analyzing *Nocardia* draft genomes, two new *Nocardia* genomes of *N. cyriacigeorgica* GUH-2 [8, 9] and *N. brasiliensis* HUJEG-1 [10] were published. We included them in the present analysis together with *N. farcinica* genome, which our group has published previously [11].

## Methods

### Strains

*N. otitidiscaviarum* IFM 11049 and *N. brasiliensis* IFM 10847 were from the IFM culture collections of MMRC, Chiba University, Japan [12]. *N. asteroides* NBRC 15531^T^ and *N. brasiliensis* NBRC 14402^T^ were from the NBRC culture collection [5]. Cells were cultured in brain heart infusion liquid culture medium (Difco) in the conventional manner.

### Acquisition of whole-genome sequences

Genomic DNA of *N. otitidiscaviarum* IFM 11049, *N. brasiliensis* (IFM 10847, NBRC 14402^T^), and *N. asteroides* NBRC 15531^T^ was prepared as described previously [13]. Genome sequences were read by the pyrosequencing method using genome sequencer GS FLX Instruments and GS FLX Titanium Kits (Roche Applied Science, Japan). The read redundancy for the four draft genomes ranged between 55 and 104. We assembled the sequence reads of *N. otitidiscaviarum* IFM 11049, *N. brasiliensis* IFM 10847, *N. brasiliensis* NBRC 14402^T^, and *N. asteroides* NBRC 15531^T^, and obtained 65, 223, 115, and 39 contigs, which were longer than 500 bp. The estimated genome sizes of *N. otitidiscaviarum* IFM 11049, *N. brasiliensis* IFM 10847, *N. brasiliensis* NBRC 14402^T^, and *N. asteroides* NBRC 15531^T^ were 7.9 Mb, 9.2 Mb, 8.9 Mb, and 7.0 Mb, respectively. The draft genome sequences of *N. otitidiscaviarum* IFM 11049, *N. brasiliensis* (IFM 10847, NBRC 14402^T^), and *N. asteroides* NBRC 15531^T^ are available at GenBank/EMBL/DDBJ under the accession numbers BATZ01000001–BATZ01000065, BAUA01000001–BAUA01000223, BAFT01000001–BAFT01000128, and BAFO01000001–BAFO01000049, respectively. The complete genome sequences of *N. cyriacigeorgica* GUH-2, *N. brasiliensis* HUJEG-1 (=ATCC 700358), and *N. farcinica* IFM 10152 were downloaded from DDBJ [14], with accession numbers FO082843, CP0033876, and AP006618, respectively.

### Analysis of PKS-I and NRPS gene clusters

The assembled contig sequences were submitted to the auto-annotation pipeline MiGAP [15, 16] at DDBJ as described previously [17]. Assigned ORFs were further searched for signature domains of PKS-I and NRPS genes using the InterPro domain database [18, 19]. ORFs having ketosynthase (KS) domain (IPR014030, IPR014031, IPR020841) or condensation (C) domain (IPR001242) were identified, and their adjacent genes were further analyzed as PKS-I and NRPS gene candidates. Module organizations were determined manually based on search results using InterPro database, results using PKS/NRPS analysis website [20], and signature sequences deduced using MOTIF search [21]. We also used antiSMASH [22, 23], a website for antibiotics and secondary metabolite analysis, for finding orthologous clusters and predicting substrates for adenylation domains. PKS-I and NRPS gene clusters of *N. farcinica* IFM 10152, *N. cyriacigeorgica* GUH-2, and *N. brasiliensis* HUJEG-1 were also identified using the *N. farcinica* genomic database [11, 24]. We assumed that two or more PKS-I and/or NRPS genes that were adjacent to each other constitute one cluster for secondary metabolite production (See Additional file 1: Table S1, for details and exceptions). We also assumed that one multi-domain PKS-I or NRPS gene that was not accompanied by adjacent PKS-I/NRPS genes constitute one independent cluster. However, genes having only a single PKS-I or NRPS domain were excluded from the present analysis because we considered them atypical, and focused on multi-domain clusters. The contig sequences containing PKS-I and NRPS gene clusters are available at GenBank/EMBL/DDBJ under the following accession numbers: [AB700569 - AB700587] (*N. otitidiscaviarum* IFM 11049), [AB701575 - AB701605] (*N. brasiliensis* IFM 10847), [AB701607 – AB701636] (*N. brasiliensis* NBRC 14402^T^), and [AB685274], [AB700124 - AB700133], [AB700557 - AB700568] (*N. asteroides* NBRC 15531^T^).

### Search for orthologous gene clusters among species and strains

BLASTP search was performed using the NCBI Protein BLAST program against the non-redundant protein sequence database [25, 26]. We considered *Nocardia* genes homologous to other genes when they have more than 70% sequence similarity in BLASTP search, and also when their domain organizations have high similarity. We also compared clusters with domain organizations that only partially match each other, as described in the text.

## Results and discussion

The two leftmost columns in Table 1 list *Nocardia* strains studied in the present paper and their exact (complete genome) or estimated (draft) genome sizes. The genome sizes ranged between 6.0 and 9.4 Mb, similar to those of representative *Streptomyces* strains (5.0 - 11.9 Mb), the most abundant sources of secondary metabolites [27–29]. The fourth column indicates that the strains are from different clinical origins.

**Table 1:** **Genome sizes and numbers of PKS-I, NRPS, and PKS-I/NRPS hybrid gene clusters in** ***Nocardia*** **strains**

Strain name	Genome size* (Mb)	State of sequence	Source	Number of gene clusters (Average number of genes/modules per cluster)			
				PKS-I	NRPS	PKS-I/NRPS hybrid	Total number of clusters
*N. farcinica* IFM 10152 [11]	6.0	Complete	Clinical (human sputum)	4	7	1	12
				(1.0/1.0)^¶^	(1.6/7.7)	(6.0/5.0)	(1.8/5.6)
*N. cyriacigeorgica* GUH-2 [8, 9]	6.2	Complete	Clinical (human kidney)	5	9	1	15
				(1.2/1.0)	(1.4/5.8)	(8.0/8.0)	(1.8/4.3)
*N. asteroides* NBRC 15531^T^[1, 30]	7.0	Draft	Clinical (fatal brain abscess)	7	13	2	22
				(1.4/2.6)	(1.2/4.5)	(4.0/4.0)	(1.5/3.9)
*N. otitidiscaviarum* IFM 11049 [31]	7.8	Draft	Clinical (human sputum)	4	12	2	18
				(1.0/1.0)	(1.2/3.3)	(4.0/11.0)	(1.5/3.6)
*N. brasiliensis* NBRC 14402^T^[32, 33]	8.9	Draft	Clinical (leg lesion)	10	13	4	27
				(1.4/1.1)	(1.6/5.0)	(5.5/4.3)	(2.1/3.4)
*N. brasiliensis* IFM 10847 [12]	9.2	Draft	Clinical (human pus)	11	13	6	30
				(1.5/1.1)	(1.5/5.1)	(4.8/4.3)	(2.2 /3.5)
*N. brasiliensis* HUJEG-1 [10]	9.4	Complete	Clinical (human mycetoma)	11	13	6	30
				(1.4/1.2)	(1.3/4.5)	(4.8/4.3)	(2.0/3.2)

*In draft genomes, genome sizes are estimated values. ^¶^Average numbers of genes/modules per cluster are indicated in parenthesis.

**Figure 1:**
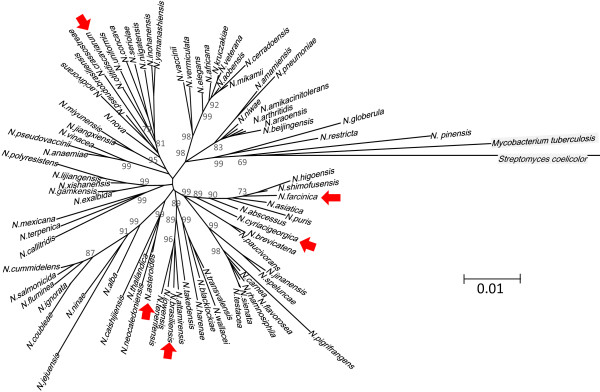
**Phylogenetic positions of** ***Nocardia*** **strains studied in the present work.** The phylogenetic tree was constructed using 16S rRNA gene sequences of type strains (http://www.bacterio.cict.fr/, http://www.ncbi.nlm.nih.gov/nuccore). MEGA5 software [34] was used to draw non-rooted neighbor-noining phylogenic tree. Positional information of *Mycobacterium tuberculosis* (Genbank accession # X58890) and *Streptomyces coelicolor* (#AB184196) was added to the tree. Bootstrap values of 1000 re-samplings are shown only for the main branches. The five species studied in the present work were marked with red arrows.

Figure 1 illustrates phylogenetic positions of the five *Nocardia* species (seven strains) studied in the present paper among 78 other established *Nocardia* strains. It also includes *Streptomyces coelicolor* and *Mycobacterium tuberculosis* for comparison. Four out of the five species are located in different clades of the 16S rRNA phylogenetic tree, indicating that the present analysis is based on information from a wide range of *Nocardia* species. *N. asteroides* and *N. cyriacigeorgica* are in the same clade. We also included three strains from *N. brasiliensis* to elucidate intra-species variations.

PKS-I, NRPS, and PKS-I/NRPS hybrid gene clusters from the *Nocardia* strains were predicted as described in Methods. Numbers of the three different types of clusters and the total number of clusters in each strain are listed in the four rightmost columns in Table 1. Among the seven strains, the numbers of PKS-I, NRPS, PKS-I/NRPS hybrid clusters, and their total number increased proportionally to the genome size, except for *N. otitidiscaviarum* and *N. asteroides* (Table 1) as reported in other genera [35]*. N. farcinica* had the least while *N. brasiliensis* HUJEG-1 had the highest number of the gene clusters. The total number of clusters within the three *N. brasiliensis* genomes differed (27 to 30), suggesting that different strains of the same species potentially produce their own unique products (see below).

We also counted the numbers of type-II PKS, type-III PKS and terpene synthesis clusters in each genome. The numbers ranged between 0 and 3, except in *N. otitidiscaviarum* and *N. brasiliensis* strains, which have five and eight clusters for terpene synthesis per genome, respectively. In the present paper, however, we focused on PKS-I and NRPS secondary metabolite clusters because their products usually have larger molecular weights with more complex chemical structures than the others and have unique pharmacological activities.

**Figure 2:**
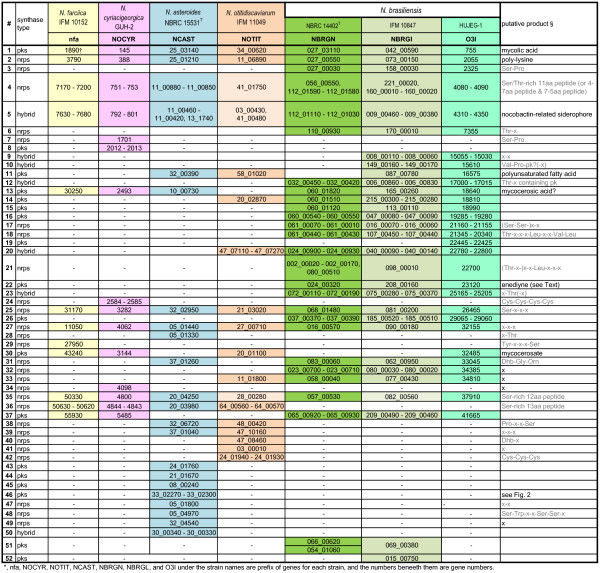
**PKS-I, NRPS, and PKS-I/NRPS hybrid gene clusters identified in genome sequences of** ***Nocardia*** **strains.**

Figure 2 shows all the clusters found in each genome. Presumptive orthologous clusters, as defined in Methods, are aligned in the same row of the table. The rightmost column shows secondary metabolites referred from the database, e.g., [23], and also those inferred using the tools described in Methods.

### Clusters common among the seven strains

Figure 2 suggests that seven presumable products (lines #1, #2, #4, #5, #25, #27, and #35) are common among the seven strains belonging to the five species. It is noteworthy that clusters #1, #2, #4, and #5 reside close to the original points of replication (ori.) in the three species whose completed genome sequences are known, in accordance with a report showing that conserved genes reside in the internal core region of actinomycete genomes [27].

#### Mycolic acid (*pks13*)

We predicted that the products of the PKS-I genes in line #1 were mycolic acids, cell wall components in members belonging to *Corynebacterineae*, because these PKS-Is showed the same domain organization as those of *pks*13 in *Mycobacterium tuberculosis* for the synthesis of mycolic acids [36], and also showed over 80% sequence similarities to PKS-Is of *N. farcinica* annotated for mycolic acid synthesis [24].

#### Poly-lysine (Pls)

NRPS genes of line #2 were predicted to be for poly-lysine synthesis because their module organizations are the same as that of poly-lysine synthetase (Pls) in *Streptomyces albulus*[37]. The corresponding gene, nfa3790, is also annotated as a Pls homolog in the *N. farcinica* database [24]. The sequence identity between Pls in *S. albulus* and nfa3790 in *N. farcinica* was 55%.

#### Ser/The-rich nonribosomal peptides

NRPS gene clusters in line #4 were present in all strains examined, but only a partial sequence was found in *N. otitidiscaviarum* (Additional file 2: Figure S1A). Zoropogui *et al.*, [9] has suggested that #4 in *N. cyriacigeorgica* was for synthesis of 2-amino-9,10 epoxi-8-oxodecanoic acid, a component of HC-toxin, which is produced by a plant pathogen causing corn leaf spots (reviewed in [38]). However, we suggest two other possibilities based on the domain organization of the cluster. One is that the intact #4 cluster is for synthesis of a serine/threonine-rich peptide composed of 11 amino acids. The second possibility is that the same sequence consists of two different NRPS clusters, since two thioesterase domains are present within the sequence, and accordingly, produces two peptide chains. In the latter case, the products of cluster #4 in *N. asteroides* would be two peptides: one composed of four amino acids (NCAST_11_00880 & NCAST_11_00870) and the other composed of seven amino acids (NCAST_11_00860 & NCAST_11_00850). Likewise, in *N. brasiliensis*, the products of cluster #4 would also be two peptides: one composed of six amino acids (O3I_004080 & O3I_004085) and the other composed of five amino acids (O3I_004090) (Figure 2, #4, rightmost column).

A possible reason why *N. otitidiscaviarum*, unlike the other species, has only a partial #4 cluster is that this species is phylogenetically distant from the other strains, as shown in Figure 1. The relationships among the products of these gene clusters, the phylogenetic positions of the strains, and their pathogenicity to plants and animals are interesting issues to clarify.

NRPS genes in line #35 were also present in all the strains examined, although those of *N. otitidiscaviarum* and *N. brasiliensis* IFM 10847 were partial compared with those of the other strains (Additional file 2: Figure S1B). The genes in five strains, except for *N. otitidiscaviarum* and *N. brasiliensis* IFM 10847, have 12 modules, and many of their adenylation domains were predicted to select Ser as the substrate. Hence, we assumed the products would be Ser-rich 12 aa peptides. Besides the clusters common among the seven strains in line #35, similar NRPS clusters were also present in the adjacent clusters of line #36, but only in four species excluding *N. brasiliensis* strains. Interestingly, the predicted products are Ser-rich 13 aa peptides.

Although the conservation of Ser- (and Thr-) rich large peptides synthesized by clusters of #4, #35, and #36 in the *Nocardia* strains suggests that they have important roles, physiological roles of the products remain to be investigated.

#### Nocobactin (*nbt*)-like siderophore

**Figure 3:**
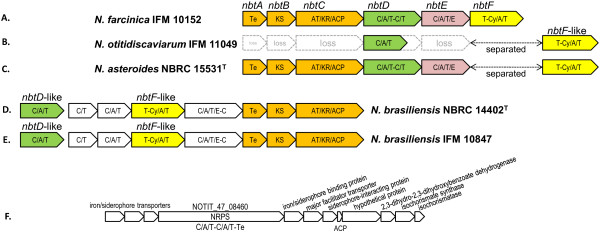
**Comparison of** ***nbt*** **-like, siderophore-synthesizing gene clusters (Figure** 2 **#20) among** ***Nocardia*** **species**. *N. farcinica* **(A)**, *N. otitidiscaviarum* **(B)**, *N. asteroides* **(C)**, *N. brasiliensis* NBRC 14402^T^ **(D)**, and *N. brasiliensis* IFM 10847 **(E)**. An *nbtF*-like gene was located distantly from other *nbt*-like genes in *N. otitiduscaviarum* and *N. asteroides* genomes. Homologous NRPSs are marked with the same colors (green, purple, and yellow), while PKS-Is are colored orange. In *N. otitidiscaviarum*, *nbtA*, *nbtB*, *nbtC*, *nbtE*-like genes were not found. **F**. Domains and module structures of a putative siderophore synthetic gene cluster (line #40 in Figure 2) found only in *N. otitidiscaviarum* IFM 11049. Putative gene functions were inferred by BLASTP search [26] and MOTIF search [21] and are indicated in the figure.

The PKS-I/NRPS hybrid cluster #5 in *N. farcinica* produces nocobactin, a siderophore and a pathogenic factor, as proven by Hoshino *et al*., [39]. Figure 3 compares clusters in line #5, which are candidates of nocobactin-like siderophore-producing genes. *N. asteroides* has a full set of genes required for siderophore synthesis (NCAST_11_00460 through NCAST_11_00420 and NCAST_13_1740 in #5). NCAST_13_1740 contains an *nbtF*-like gene [39], but is found separated from the rest of *nbt* genes in contig 11 by more than 180 Mb based on the contig sequences. In *N. brasiliensis,* the corresponding cluster structure is different from that in *N. farcinica* in terms of module number and domain organization, suggesting that a functionally similar but structurally different molecule is synthesized in *N. brasiliensis*. In *N. otitidiscaviarum*, cluster #5 lacks genes corresponding to *nbtA-C* and *nbtE*, which are required for nocobactin synthesis in *N. farcinica*[39], suggesting that cluster #5 in *N. otitidiscaviarum* may not be able to produce a nocobactin-like siderophore. Interestingly, however, *nbtD*- and *nbtF*-like genes, which both have 75% similarities to *nbtD* and *nbtF* genes in *N. farcinica*, respectively, are found in the middle of two different contigs in the *N. otitidiscaviarum* genome (contigs 3 and 41, respectively; listed in cluster #5 in Figure 2; see also Figure 3), suggesting gene loss/gain and recombination during evolution within the genus *Nocardia*. Such gene loss/gain is not only observed in the *nbt*-like gene cluster #5, but also found in nfa7170-7200 homologs (#4), nfa50330 homologs (cluster #35), and nfa50630-50620 homologs (#36), as shown in Additional file 2 (Figure S1). It is further noteworthy that *N. otitidiscaviarum* has another candidate gene cluster for siderophore synthesis, which is shown in line #40 of Figure 2. The domain and module organization of this cluster are shown in Figure 3F, which shows the differences from the one for nocobactin synthesis.

#### Other common gene clusters

Two NRPS genes in lines #25 and #27 (Figure 2) were common in all the strains sequenced, having four and three modules, respectively. However, the amino-acid composition of the products could not be predicted *in silico* using antiSMASH. Chemical structures and physiological roles of the products remain to be investigated.

### Clusters missing in a few strains but found in others

#### Mycocerosic acid

PKS-I clusters of line #13 (Figure 2) were present in the six strains except *N. otitidiscaviarum*. Other PKS-I clusters in line #30 were present in the four strains but not in *N. asteroides* and in the two *N. brasiliensis* strains (NBRC 14402^T^, IFM 10847). Nfa30250 (Figure 2 #13) has been predicted to be involved in mycocerosic acid synthesis in the *N. farcinica* genome project [24]. On the other hand, O3I_032485 in *N. brasiliensis* HUJEG-1 (Figure 2 #30) has been putatively annotated as mycocerosate synthase [10]. Both PKS-Is in *N. farcinica* (#13) and in *N. brasiliensis* (#30) showed approximately 47% amino acid similarities to *Mycobacterium tuberculosis* PKS-I [GenBank/EMBL/DDBJ accession number: CCP46654] for the synthesis of mycocerosic acid, a pathogenic factor [40, 41]. All the PKS-Is listed in line #13 and #30 showed sequence similarities ranging between 62 and 75%, having almost the same protein length (approximately 2200 aa) and identical domain organization (KS/AT/DH/KR/ER/ACP) (Additional file 1: Table S1).

It is possible that in *N. otitidiscaviarum*, cluster #30 is a substitute for cluster #13. Interestingly, *N. farcinica* and *N. cyriacigeorgica* possess both #13 and #30, which could be related to the strong pathogenicity of the two strains.

#### Polyunsaturated fatty acid (*pfaA*)

Orthologous genes of cluster #11 are found in *N. asteroides*, *N. otitidiscaviarum*, and *N. brasiliensis* IFM 10487 and HUJEG-1, but not in *N. farcinica*, *N. cyriacigeorgica*, and *N. brasiliensis* NBRC 14402 (Figure 2). The PKS-I sequence identities among *N. asteroides*, *N. otitidiscaviarum*, and *N. brasiliensis* ranged from 66 to 78%. The modular structures of PKS-Is in line #11 (KS/AT/ACP/ACP/KR) are unusual because they have two tandem ACP domains, and one KR domain is located after ACP (Additional file 3: Figure S2). These features have been known in polyunsaturated fatty acid (PUFA) synthase, PfaA, of marine bacteria, such as those belonging to the genus *Shewanella*[42, 43]. The module organizations are similar between #11 and PfaA (Figure S2), and their amino-acid sequence similarities are over 50%. Hence, we predict that the products of PKS-I in line #11 are polyunsaturated fatty acids, as already reported in *N. brasiliensis* HUJEG-1 genome [10]. However, reports of production of polyunsaturated fatty acids have been limited only in some psychrophilic, piezophilic, or halophilic bacteria in prokaryotes [44, 45] and have not been found in *Nocardia* strains. Thus, future chemical and synthetic analysis are required to explore the potential of *Nocardia* strains as industrial producers of these pharmaceutically and nutraceutically valuable compounds.

### Species-specific clusters

**Figure 4:**
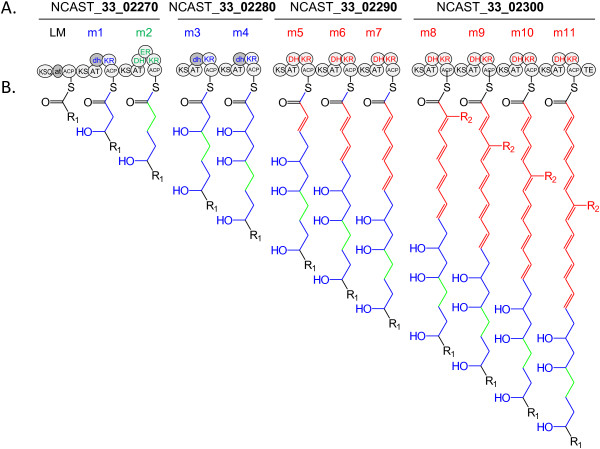
**A representative example of PKS-I gene cluster (#46 in Figure** 2 **) (A), and its putative product (B).** The cluster is specific to *N. asteroides* NBRC 15531^T^. **A**. Domain organization. LM, loading module; m1 – m11, modules; KS, ketosynthase; AT, acyltransferase; ACP, acyl carrier protein; DH, dehydratase; KR, ketoreductase; ER, enoyl-reductase domains. Gray ’DH” domains are probably inactive. **B**. Chemical structure of intermediate polyketide chain predicted from the gene cluster #46 based on assembly line rule [46, 49].

Only a few species-specific clusters are found in *N. farcinica* (cluster #29), *N. cyriacigeorgica* (#7, #8, #24, #34), and *N. otitidiscaviarum* (#40, #41, #42), suggesting that the evolution of their polyketides and nonribosomal metabolites are not as dynamic as in other species (Figure 2). On the other hand, *N. asteroides* has nine species-specific clusters (PKS-I, #43 - #46; NRPS, #28, #47 - #49; PKS-I/NRPS hybrid, #50). Among them, we selected #46 as a representative unique cluster structure in *N. asteroides* (Figure 4A). The cluster consists of four adjacent genes, namely, NCAST33_02270, _02280, _02290, and _02300, each of which has the top BLAST-hit to a gene sequence in different *Streptomyces* strains (see Additional file 1: Table S1, for more details). The cluster consisted of twelve modules and was larger than any other cluster in the *Nocardia* strains studied in this paper. The orders of domains within the modules follow the accepted theory for PKS-I gene cluster structures, i.e., having repeats of “KS-AT-optional domains-ACP” [46, 47]. The same module organization, however, could not be found in available public databases, and the cluster has an amino-acid sequence similarity of less than 53% to any other known PKS-I clusters. This suggests that the gene cluster may be involved in production of a novel secondary metabolite. We propose a chemical structure of the polyketide chain synthesized by this cluster as shown in Figure 4B based on the PKS-I assembly line rule [46] as follows. The presence of a ketosynthaseQ (KSQ) domain at the N terminus of NCAST_33_02270 and a thioesterase (Te) domain at the C terminus of NCAST_33_02300 indicates that NCAST_33_02270 and NCAST_33_02300 contain the modules that initiate and terminate PKS-I assembly line, respectively. Among the eleven AT domains of module 1 (m1) to 11 (m11), ten AT domains, except that in m8, had the HAFHS signature amino-acid sequence, which is specific for malonyl-CoA in substrate recognition [48, 49]. The substrate of the AT domain in m8, which has IASHS amino-acid sequence, and the starter molecules loaded on LM could not be predicted using bioinformatic approach. Hence, this polyketide backbone is predicted to be Cx-C_2_-C_2_-C_2_-C_2_-C_2_-C_2_-C_2_-Cy-C_2_-C_2_-C_2,_ where C_2_ is a unit derived from malonyl-CoA, and Cx and Cy are carbon backbones derived from presently unknown substrates. An inactive DH (dh)-KR pair, which is responsible for formation of a hydroxyl group, is present as an optional domain in m1, m3, and m4 modules, while the optional domain present in m2 is a DH-ER-KR trio, which completely reduces the ketone residue formed by the m2 module. Optional domains of m5 through m11 are DH-KR pairs, which form double bonds from the ketone residues produced by these modules. The resulting molecule has a polyketide backbone consisting of more than 23 carbons.

The *N. brasiliensis* strains had the largest number of species-specific clusters among the strains studied in the present work: five to six PKS-I clusters (#15, #16, #19, #22, #26, #51, #52), six NRPS clusters (#3, #6, #17, #18, #21, #32), and two to four PKS-I/NRPS hybrid clusters (#9, #10, #12, #23), depending on the strains. Among them, three PKS-I clusters (#22, #51, #52) consisted of a single module, suggesting that their final or intermediate products may be small in accordance with the assembly line rule [46, 47], unless the modules are used iteratively, as has been reported in actinomycetes (e.g., [50, 51]). Interestingly, PKS-Is in #22 shows 61% sequence similarity to PksE of *Streptomyces griseus* [GenBank/EMBL/DDBJ accession number: AAO25858], whose product is an unusual polyketide compound including a 9-membered enediyne core [52].

The rest of the clusters with multiple modules may possibly produce large species-specific products. In particular, nonribosomal peptides produced by clusters #18 and #21 are, respectively, predicted to consist of nine and six (to eight) amino acids.

### Intra-species variations of clusters

Strain-specific as well as species-specific PKS-I clusters were found in *N. brasiliensis* strains (#19, #30, #52), indicating different strains of the same species may potentially produce different products. Because there are large numbers of *Nocardia* strains stored in several bio-resource centers (e.g., [5–7]), and are rapidly accumulating [12], these *Nocardia* strains constitute a highly promising future resource for exploring secondary metabolites.

### Other unique examples of clusters

**Figure 5:**
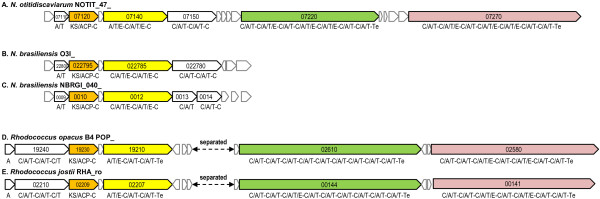
**Large PKS-I/NRPS hybrid gene clusters found in** ***N. otitidiscaviarum*** **,** ***N. brasiliensis*** **HUJEG-1 and IFM 10847 (Figure** 2 **#20). A**; The hybrid gene cluster found in *N. otitidiscaviarum*. **B** and **C**; *N. brasiliensis* HUJEG-1 and IFM 10847 have only the left half of the hybrid cluster shown in **A**. A similar cluster in *N. brasiliensis* NBRC 14402^T^ is truncated at an edge of a contig, and not shown here. **D** and **E**; Clusters similar to the one in A are found in *Rhodococcus opacus* B4 (D) [53], and *Rhodococcus jostii* RHA1 (E) [54]. Loci of the left clusters and the right two NRPS clusters are separated in *Rhodococcus* genomes.

Figure 5A shows a module structure of PKS-I/NRPS hybrid cluster #20 (Figure 2) in *N. otitidiscaviarum*. The left half (approximately 8,300 aa) of the cluster has high similarity to *N. brasiliensis* hybrid cluster #20 (75 - 82% similarities), but the right half (7,746 aa of NOTIT_47_07220 plus 9,157 aa of NOTIT_47_07270) has top hits to two proteins in *Rhodococcus opacus* (7,746 aa) [GenBank/EMBL/DDBJ accession number: YP_002777453] (Figure 5D) and *Gordonia aichiensis* (9,517 aa) [GenBank/EMBL/DDBJ accession number: WP_005170336], with 59% (4,667/7,781) and 54% (4,543/8,322) amino acid similarities, respectively. The right-half consisting of two NRPSs is widely conserved in *Rhodococcus* spp. including *R. jostii* as shown in Figure 5E.

Because the gene cluster of *N. otitidiscaviarum* #20 contains one PKS-I module and 19 NRPS modules, the product has been tentatively predicted to include one polyketide chain and 19 amino acids (Additional file 4: Figure S4). It should also be mentioned, however, that the two genes in cluster #20, NOTIT_47_07270 and NOTIT_47_07220, each contain thioesterase domains (Te) at their C-terminal ends, suggesting another possibility that the product may contain 12 amino acids instead of 19.

All cluster structures we analyzed in the present work are listed in (Additional file 1: Table S1).

## Conclusions

We conclude the following: 1) genomes of *Nocardia* strains carry as many PKS-I and NRPS clusters as *Streptomyces* strains, 2) the number of PKS-I and NRPS gene clusters in *Nocardia* strains varies substantially depending on species, and *N. brasiliensis* strains carry the largest number of clusters among the species studied, 3) the seven *Nocardia* strains studied in the present work have six common PKS-I/NRPS clusters, some of whose products are yet to be studied, and 4) different *N. brasiliensis* strains have a few different clusters for secondary metabolite synthesis. Also, the following are suggested: 1) there is no clear relation between genome size and pathogenicity in *Nocardia* strains, e.g. *N. farcinica* and *N. brasiliensis* are both prevalent pathogens, but their genome sizes are 6.0 Mb and 9.4 Mb, respectively, the minimum and maximum among the strains studied, and 2) some genes (e.g. cluster #17 in Figure 2) are likely to have been horizontally transferred from (or to) other actinomycetous strains, such as *Rhodococcus* spp.

To summarize, in this study, we compared complete and draft genome sequences of seven strains from five representative *Nocardia* species. The sequences we obtained provided useful information for inferring numbers and molecular structures of secondary metabolites potentially produced by *Nocardia* strains. Genome sequencing revealed the possibility that *Nocardia* strains are as attractive resources as *Streptomyces* strains, the largest resource of natural compounds, in the search for new useful secondary metabolites.

## Authors’ information

HK: Senior Chief, Biological Resource Center, National Institute of Technology and Evaluation (NBRC).

NI: Chief, NBRC.

AH: Senior Chief, NBRC.

AT-N: Postdoctoral Fellow, Medical Mycology Research Center (MMRC), Chiba University.

TM: Research Associate, MMRC, Chiba University.

KS: Senior Director, NBRC.

NF: Senior Director, NBRC.

TG: Professor, MMRC, Chiba University.

## Electronic supplementary material

Additional file 1: Table S1: ORFs and module/domain structures of PKS-I, NRPS, and PKS-I/NRPS hybrid gene clusters in genomes. Data for *N. otitidiscaviarum* IFM 11049, *N. asteroides* NBRC 15531 T, *N. brasiliensis* NBRC 14402 T and IFM 10847 are shown. (XLSX 33 KB)

Additional file 2: Figure S1: Representative NRPS gene clusters in *N. farcinica* and their homologs in other strains. A. *N. asteroides* has a cluster with an overall similarity to nfa7170-7200; but the third ORF, NCAST_11_00860, is similar to the first ORF nfa7170, rather than the third ORF nfa7190 of the corresponding position. *N. brasiliensis* NBRC 14402^T^ and IFM 10847 lack ORFs corresponding to nfa7180. B. *N. brasiliensis* IFM 10847 has only partial sequences *of N. farcinica* nfa50330-homologous gene, while the homolog in *N. otitidiscaviarum is* not only partial but also distantly located in the genome. C. *N. asteroides* possesses an nfa50630 homolog, but lacks an nfa50620 homolog. *N. brasiliensis* strains have no homologs. (PPTX 105 KB)

Additional file 3: Figure S2: Comparison of putative polyunsaturated fatty acid synthase (PfaA) genes between the genus *Nocardia* (Figure 2 #11) and the genus *Shewanella.* (PPTX 76 KB)

Additional file 4: Figure S3: Predicted chemical structure of the product from PKS-I/NRPS hybrid gene cluster #20 in *N. otitidiscaviarum.* (PPTX 107 KB)

## Abbreviations

PKS: Polyketide synthase
PKS-I: Type-I polyketide synthase
NRPS: Nonribosomal peptide synthetase
NBRC: Biological Resource Center, National Institute of Technology and Evaluation
MMRC: Medical Mycology Research Center
KS: Ketosynthase
AT: Acyltransferase
ACP: Acyl carrier protein
KR: Ketoreductase
Te: Thioestarase
LM: Loading module
DH: Dehydratase
dh: Inactive dehydratase
ER: Enoyl-reductase.
