# Supplementary material for: Genome based analysis of type-I polyketide synthase and nonribosomal peptide synthetase gene clusters in seven strains of five representative Nocardia species
Source: BMC Genomics. 2014 Apr 30;15(1):323. doi: 10.1186/1471-2164-15-323 (PMC4035055; doi:10.1186/1471-2164-15-323)
Supplement: Supplementary file 2 — Additional file 2: Figure S1: Representative NRPS gene clusters in N. farcinica and their homologs in other strains. A. N. asteroides has a cluster with an overall similarity to nfa7170-7200; but the third ORF, NCAST_11_00860, is similar to the first ORF nfa7170, rather than the third ORF nfa7190 of the corresponding position. N. brasiliensis NBRC 14402T and IFM 10847 lack ORFs corresponding to nfa7180. B. N. brasiliensis IFM 10847 has only partial sequences of N. farcinica nfa50330-homologous gene, while the homolog in N. otitidiscaviarum is not only partial but also distantly located in the genome. C. N. asteroides possesses an nfa50630 homolog, but lacks an nfa50620 homolog. N. brasiliensis strains have no homologs. (PPTX 105 KB) [file 12864_2013_6019_MOESM2_ESM.pptx]

## Slide 1
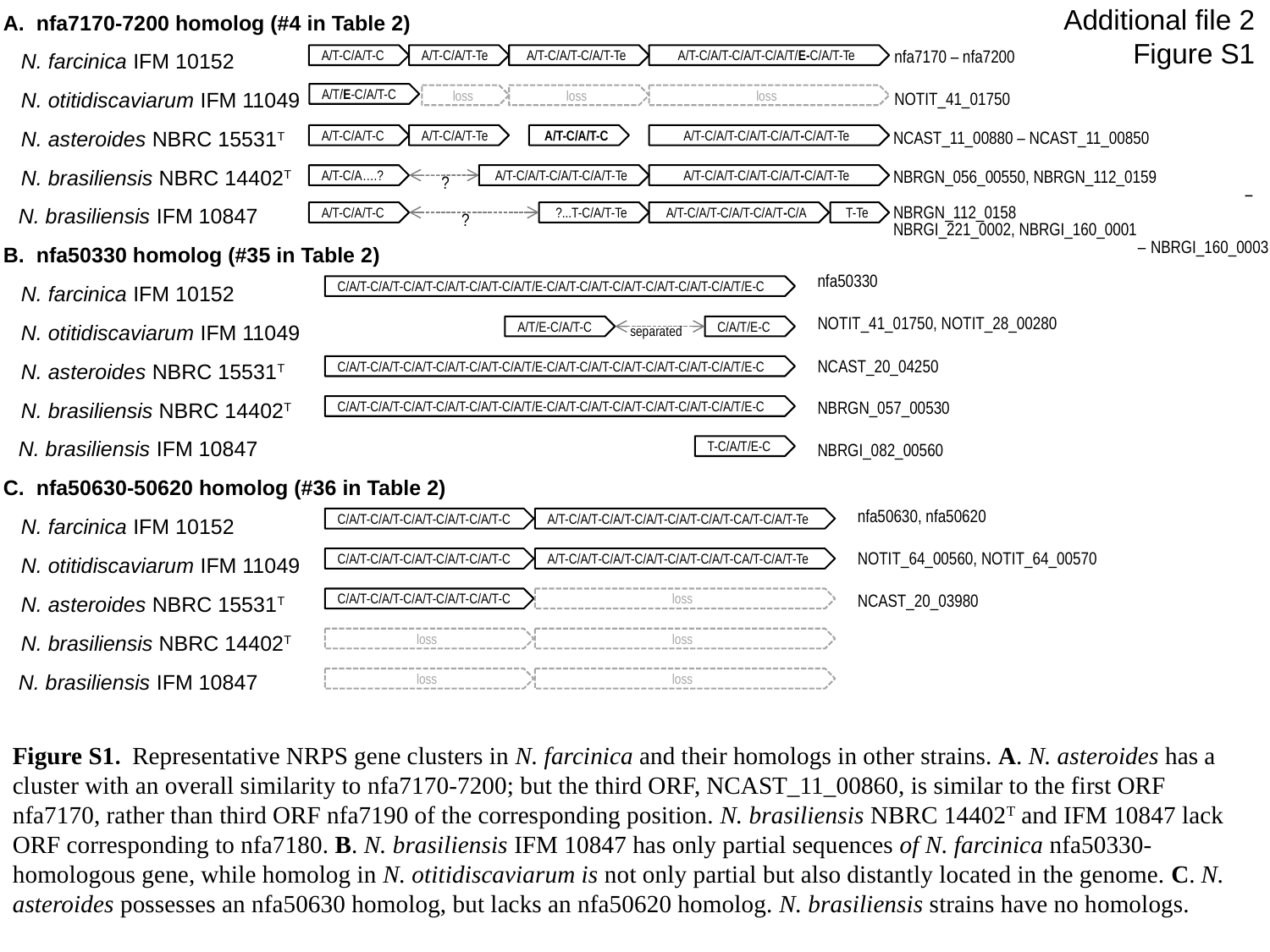

A. nfa7170-7200 homolog (#4 in Table 2)
 N. farcinica IFM 10152
 N. otitidiscaviarum IFM 11049
 N. asteroides NBRC 15531T
 N. brasiliensis NBRC 14402T
 N. brasiliensis IFM 10847
B. nfa50330 homolog (#35 in Table 2)
 N. farcinica IFM 10152
 N. otitidiscaviarum IFM 11049
 N. asteroides NBRC 15531T
 N. brasiliensis NBRC 14402T
 N. brasiliensis IFM 10847
C. nfa50630-50620 homolog (#36 in Table 2)
 N. farcinica IFM 10152
 N. otitidiscaviarum IFM 11049
 N. asteroides NBRC 15531T
 N. brasiliensis NBRC 14402T
 N. brasiliensis IFM 10847
# Additional file 2 Figure S1
nfa7170 – nfa7200
NOTIT_41_01750
A/T-C/A/T-C
A/T-C/A/T-Te
A/T-C/A/T-C/A/T-Te
A/T-C/A/T-C/A/T-C/A/T/E-C/A/T-Te
A/T/E-C/A/T-C
loss
loss
loss
NCAST_11_00880 – NCAST_11_00850
NBRGN_056_00550, NBRGN_112_0159
　　　　　　　　　　　　　　　　　　　　– NBRGN_112_0158
NBRGI_221_0002, NBRGI_160_0001
 – NBRGI_160_0003
A/T-C/A/T-C
A/T-C/A/T-Te
A/T-C/A/T-C
A/T-C/A/T-C/A/T-C/A/T-C/A/T-Te
?
A/T-C/A/T-C/A/T-C/A/T-Te
A/T-C/A/T-C/A/T-C/A/T-C/A/T-Te
A/T-C/A….?
A/T-C/A/T-C
?
?...T-C/A/T-Te
A/T-C/A/T-C/A/T-C/A/T-C/A
T-Te
nfa50330
NOTIT_41_01750, NOTIT_28_00280
NCAST_20_04250
NBRGN_057_00530
NBRGI_082_00560
C/A/T-C/A/T-C/A/T-C/A/T-C/A/T-C/A/T/E-C/A/T-C/A/T-C/A/T-C/A/T-C/A/T-C/A/T/E-C
separated
A/T/E-C/A/T-C
C/A/T/E-C
C/A/T-C/A/T-C/A/T-C/A/T-C/A/T-C/A/T/E-C/A/T-C/A/T-C/A/T-C/A/T-C/A/T-C/A/T/E-C
C/A/T-C/A/T-C/A/T-C/A/T-C/A/T-C/A/T/E-C/A/T-C/A/T-C/A/T-C/A/T-C/A/T-C/A/T/E-C
T-C/A/T/E-C
nfa50630, nfa50620
NOTIT_64_00560, NOTIT_64_00570
NCAST_20_03980
C/A/T-C/A/T-C/A/T-C/A/T-C/A/T-C
A/T-C/A/T-C/A/T-C/A/T-C/A/T-C/A/T-CA/T-C/A/T-Te
C/A/T-C/A/T-C/A/T-C/A/T-C/A/T-C
A/T-C/A/T-C/A/T-C/A/T-C/A/T-C/A/T-CA/T-C/A/T-Te
C/A/T-C/A/T-C/A/T-C/A/T-C/A/T-C
loss
loss
loss
loss
loss
Figure S1. Representative NRPS gene clusters in N. farcinica and their homologs in other strains. A. N. asteroides has a cluster with an overall similarity to nfa7170-7200; but the third ORF, NCAST_11_00860, is similar to the first ORF nfa7170, rather than third ORF nfa7190 of the corresponding position. N. brasiliensis NBRC 14402T and IFM 10847 lack ORF corresponding to nfa7180. B. N. brasiliensis IFM 10847 has only partial sequences of N. farcinica nfa50330-homologous gene, while homolog in N. otitidiscaviarum is not only partial but also distantly located in the genome. C. N. asteroides possesses an nfa50630 homolog, but lacks an nfa50620 homolog. N. brasiliensis strains have no homologs.
