# Supplementary material for: Genome based analysis of type-I polyketide synthase and nonribosomal peptide synthetase gene clusters in seven strains of five representative Nocardia species
Source: BMC Genomics. 2014 Apr 30;15(1):323. doi: 10.1186/1471-2164-15-323 (PMC4035055; doi:10.1186/1471-2164-15-323)
Supplement: Supplementary file 3 — Additional file 3: Figure S2: Comparison of putative polyunsaturated fatty acid synthase (PfaA) genes between the genus Nocardia (Figure 2 #11) and the genus Shewanella. (PPTX 76 KB) [file 12864_2013_6019_MOESM3_ESM.pptx]

## Slide 1
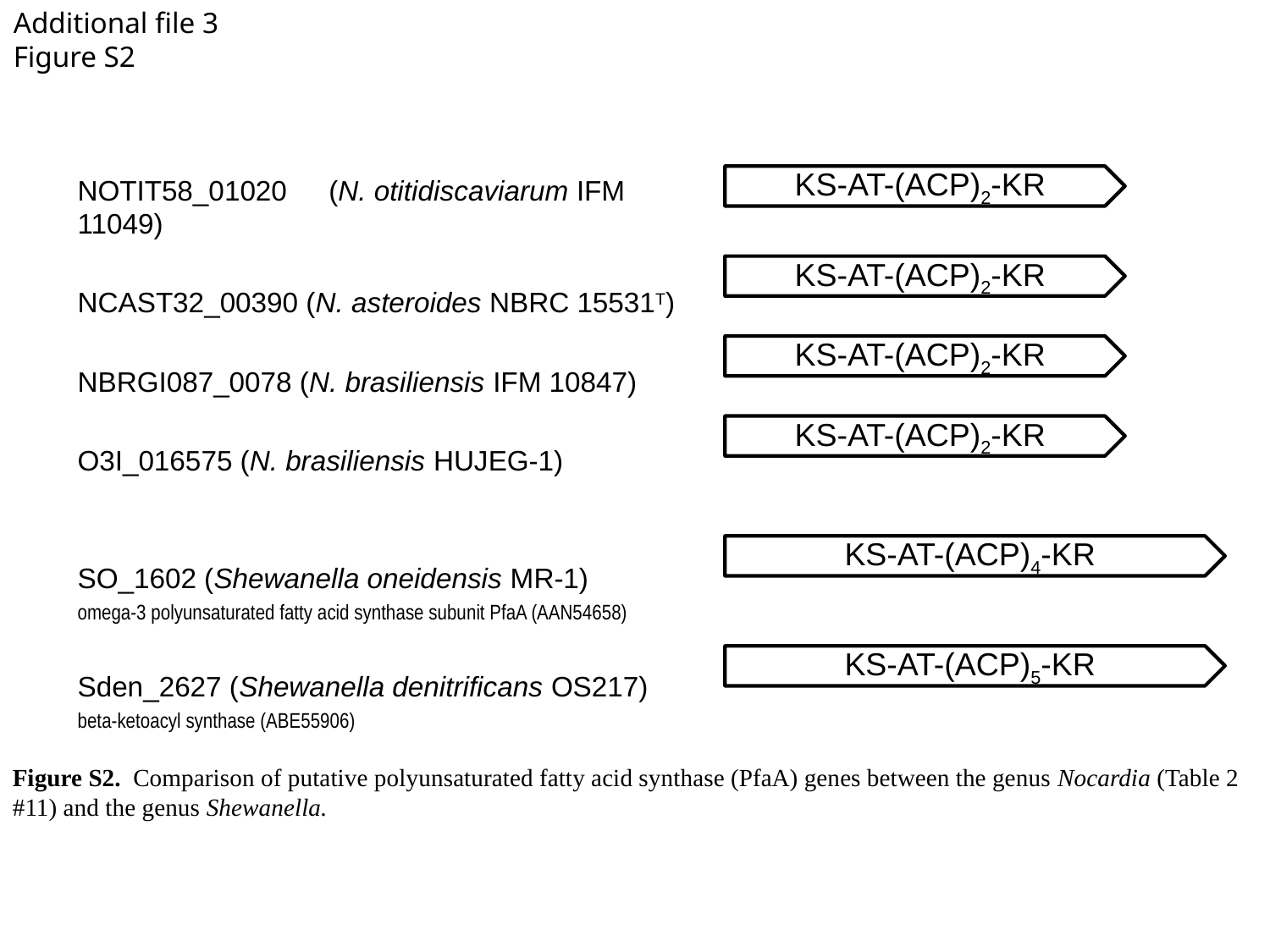

# Additional file 3 Figure S2
NOTIT58_01020　(N. otitidiscaviarum IFM 11049)
NCAST32_00390 (N. asteroides NBRC 15531T)
NBRGI087_0078 (N. brasiliensis IFM 10847)
O3I_016575 (N. brasiliensis HUJEG-1)
SO_1602 (Shewanella oneidensis MR-1)
omega-3 polyunsaturated fatty acid synthase subunit PfaA (AAN54658)
Sden_2627 (Shewanella denitrificans OS217)
beta-ketoacyl synthase (ABE55906)
KS-AT-(ACP)2-KR
KS-AT-(ACP)2-KR
KS-AT-(ACP)2-KR
KS-AT-(ACP)2-KR
KS-AT-(ACP)4-KR
KS-AT-(ACP)5-KR
Figure S2. Comparison of putative polyunsaturated fatty acid synthase (PfaA) genes between the genus Nocardia (Table 2 #11) and the genus Shewanella.
