# Supplementary material for: Genome based analysis of type-I polyketide synthase and nonribosomal peptide synthetase gene clusters in seven strains of five representative Nocardia species
Source: BMC Genomics. 2014 Apr 30;15(1):323. doi: 10.1186/1471-2164-15-323 (PMC4035055; doi:10.1186/1471-2164-15-323)
Supplement: Supplementary file 4 — Additional file 4: Figure S3: Predicted chemical structure of the product from PKS-I/NRPS hybrid gene cluster #20 in N. otitidiscaviarum. (PPTX 107 KB) [file 12864_2013_6019_MOESM4_ESM.pptx]

## Slide 1
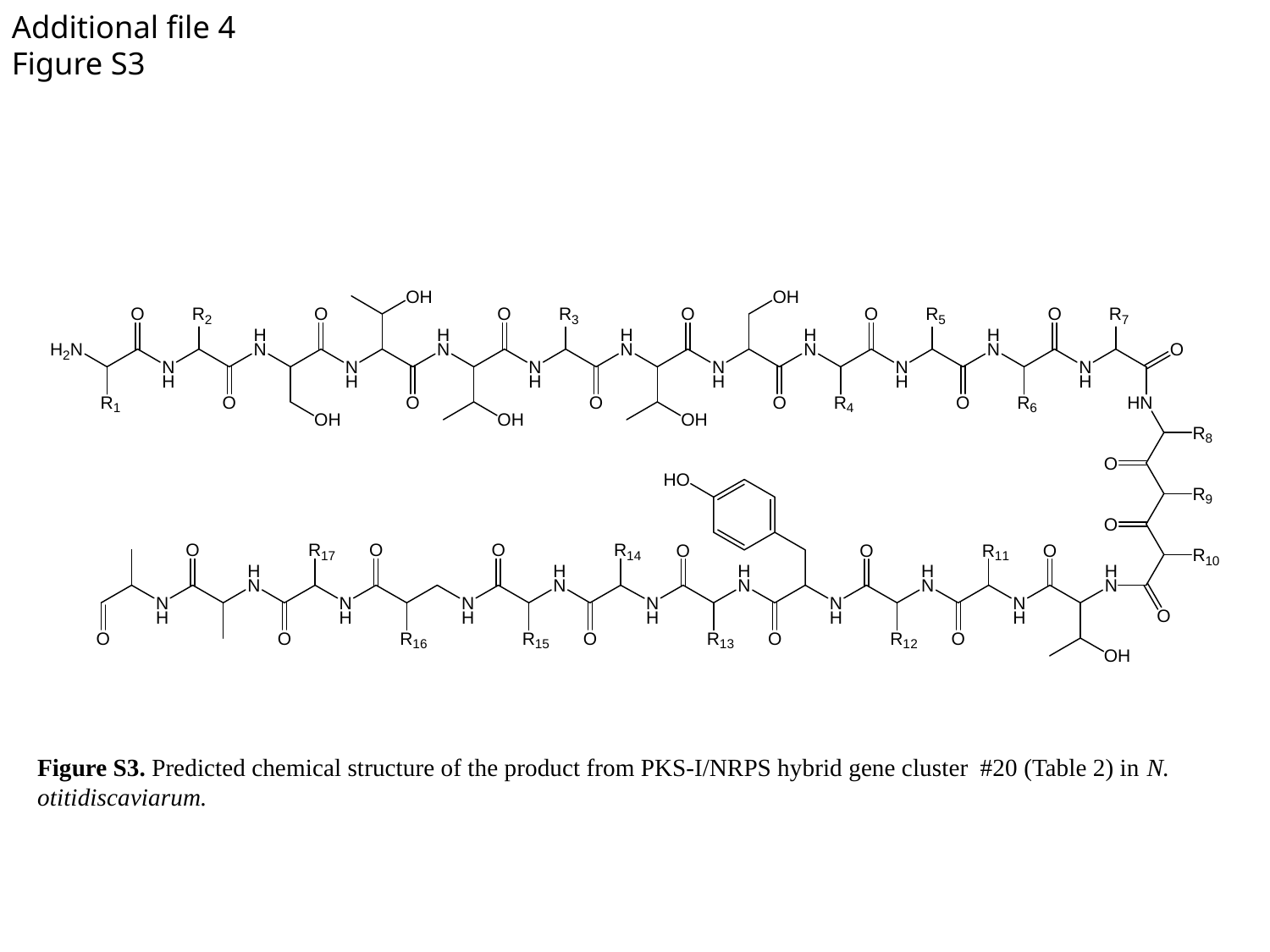

# Additional file 4 Figure S3
Figure S3. Predicted chemical structure of the product from PKS-I/NRPS hybrid gene cluster #20 (Table 2) in N. otitidiscaviarum.
